# Corrosion-resistant single-atom catalysts for direct seawater electrolysis

**DOI:** 10.1093/nsr/nwaf060

**Published:** 2025-02-22

**Authors:** Yue Zhang, Weikang Wan, Yudi Peng, Yujun Guo, Jialing Zhou, Shengchen Wang, Jiayao Yuan, Yuru Liao, Linsheng Liu, Yifan Zhang, Suli Liu, Dingsheng Wang, Zhihui Dai

**Affiliations:** School of Chemistry and Molecular Engineering, Nanjing Tech University, Nanjing 211816, China; School of Chemistry and Molecular Engineering, Nanjing Tech University, Nanjing 211816, China; School of Chemistry and Molecular Engineering, Nanjing Tech University, Nanjing 211816, China; School of Chemistry and Molecular Engineering, Nanjing Tech University, Nanjing 211816, China; School of Chemistry and Molecular Engineering, Nanjing Tech University, Nanjing 211816, China; School of Chemistry and Molecular Engineering, Nanjing Tech University, Nanjing 211816, China; School of Chemistry and Molecular Engineering, Nanjing Tech University, Nanjing 211816, China; School of Chemistry and Molecular Engineering, Nanjing Tech University, Nanjing 211816, China; School of Chemistry and Molecular Engineering, Nanjing Tech University, Nanjing 211816, China; School of Chemistry and Molecular Engineering, Nanjing Tech University, Nanjing 211816, China; School of Chemistry and Molecular Engineering, Nanjing Tech University, Nanjing 211816, China; Department of Chemistry, Tsinghua University, Beijing 100084, China; School of Chemistry and Molecular Engineering, Nanjing Tech University, Nanjing 211816, China

**Keywords:** seawater electrolysis, corrosion resistance, doping engineering, protective layer, *in-situ* characterization

## Abstract

Direct seawater electrolysis (DSE) for hydrogen production is an appealing method for renewable energy storage. However, DSE faces challenges such as slow reaction kinetics, impurities, the competing chlorine evolution reaction at the anode, and membrane fouling, making it more complex than freshwater electrolysis. Therefore, developing catalysts with excellent stability under corrosion and fulfilling activity is vital to the advancement of DSE. Single-atom catalysts (SACs) with excellent tunability, high selectivity and high active sites demonstrate considerable potential for use in the electrolysis of seawater. In this review, we present the anodic and cathodic reaction mechanisms that occur during seawater cracking. Subsequently, to meet the challenges of DSE, rational strategies for modulating SACs are explored, including axial ligand engineering, carrier effects and protective layer coverage. Then, the application of *in-situ* characterization techniques and theoretical calculations to SACs is discussed with the aim of elucidating the intrinsic factors responsible for their efficient electrocatalysis. Finally, the process of scaling up monoatomic catalysts for the electrolysis of seawater is described, and some prospective insights are provided.

## Teaser

This review explores the groundbreaking potential of corrosion-resistant single-atom catalysts in enabling efficient and sustainable hydrogen production through direct seawater electrolysis.

## INTRODUCTION

Hydrogen produced through electrolysis represents a promising alternative energy source, offering high purity, high energy density and the absence of emissions [1–3]. Also, it exerts vital effects with regard to improving environmental quality [4,5]. However, the production of hydrogen from electrolytic water is hindered by a shortage of freshwater resources. Additionally, the lack of budget and highly active and stable catalysts hinders the industrial application of water electrolysis [6,7]. Researchers attempted to find a more cost-effective method of producing hydrogen by exploring alternatives to freshwater [8,9]. Seawater, one of the most abundant and easily accessible natural water sources on Earth, is estimated to make up 96.5% of the world's water resources [10]. A review of historical reports indicates that the capital costs of desalination and purification systems have been significantly lower than those of electrolytic water systems [11]. However, recent analyses suggest that this may be changing due to rapid advances in electrolyzer design and electrolysis technology. For medium-sized systems (with a capacity range of 12 000–60 000 m^3^/day), desalination costs range from US$0.44/m^3^ to US$1.62/m^3^. In comparison, the cost of alkaline electrolyzers has consistently decreased to US$200 per kilowatt or less, driven by advancements in design [10,12]. Furthermore, due to streamlined equipment, direct seawater electrolysis (DSE) is better suited to producing hydrogen in space-constrained offshore or mobile marine systems [13].

Therefore, the development of electrolytic seawater hydrogen production technology is of great importance as it allows the use of rich seawater resources [14]. Nevertheless, the process of seawater electrolysis for hydrogen production continues to present significant challenges in comparison to the conventional method of freshwater electrolysis. In the cathodic hydrogen evolution reaction (HER), the most significant challenge is the presence of a variety of dissolved cations (such as Na^+^, Mg^2+^ and Ca^2+^), bacteria/micro-organisms and impurities like small particles [15,16]. Such contaminants can obstruct the electrodes during seawater electrolysis, and the poisoning or accelerated ageing of the electrodes/catalysts in the electrolysis system results in a reduction in the durability of the system [17–19]. Typically, the competitive and side chloride evolution reaction (CER) can impede the oxygen evolution reaction (OER) at the anode [20–22]. Previous research has demonstrated that the formation of hypochlorite is kinetically more advantageous than the four-electron OER [23–26]. Consequently, the development of highly selective and stable catalysts in DSE is necessary.

It is widely accepted that noble metals (such as Pt) and noble metal oxides (such as RuO_2_ and IrO_2_) represent efficient electrocatalysts for the HER and OER, respectively [27]. However, their capabilities in seawater electrolysis are significantly lower than in freshwater electrolytes. More importantly, the high cost and scarcity of noble metals severely limit their mass application [28]. Therefore, it is crucial to decrease the use of noble metals while maintaining high activity in practical applications [29]. In recent years, due to the high atom utilization [30], unique electronic structure and strong metal-supported interactions [31,32], single-atom catalysts (SACs), characterized by the presence of isolated metal atoms as active sites, have emerged as a transformative innovation in catalysis [33–35]. Compared to traditional multi-atom catalysts, SACs demonstrate nearly perfect atomic utilization, maximizing the efficiency of each catalytic site [36,37]. This distinctive feature positions SACs as highly efficient materials for energy-related applications, particularly in seawater catalysis, where efficient and durable catalysts are critical. One of the remarkable advantages of SACs lies in their large specific surface area, which facilitates a higher exposure of active sites [38]. This not only enhances the accessibility of reactants but also accelerates reaction rates, resulting in superior catalytic performance [39,40]. Additionally, the reduced catalyst loading associated with SACs makes them more environmentally friendly and cost-effective. The tunable electronic structure of single metal atoms in SACs further amplifies their versatility [41]. By tailoring the carrier material, coordination environment or electronic interactions with the substrate, researchers can fine-tune the activity and selectivity of SACs, adapting them for specific reactions under complex marine conditions, therefore in seawater catalysis, SACs exhibit exceptional stability and resistance to deactivation [40–42]. Unlike conventional catalysts prone to aggregation, poisoning or degradation under harsh conditions, the monoatomic nature of SACs minimizes these issues. Their unique structure reduces the adsorption of impurity ions such as chloride (Cl^−^) from seawater, thereby maintaining catalytic selectivity and stability over prolonged operations [38]. Furthermore, SACs can withstand the high salinity, variable temperatures and pressure conditions of marine environments, making them particularly well-suited for seawater electrolysis and other ocean energy applications [38].

From an economic perspective, the theoretical atom utilization rate of SACs can approach 100%, significantly reducing material costs while enhancing energy efficiency [35]. This positions SACs as a viable solution for addressing the cost and scalability challenges of large-scale seawater energy conversion technologies, such as hydrogen production [34]. Beyond practical applications, studying the reaction mechanisms and dynamic behavior of SACs in seawater catalysis can provide valuable insights into the coupling between catalyst properties and the complex chemical parameters of the marine environment [36]. This knowledge will lay the groundwork for designing SACs tailored to large-scale energy production and resource recovery in oceans [33,39]. SACs not only represent a new frontier in catalysis with their unprecedented atomic efficiency but also hold immense potential for revolutionizing seawater catalysis [41]. By bridging fundamental research with practical applications, SACs pave the way for innovative solutions in sustainable energy conversion and ocean resource development.

**Figure 1.**
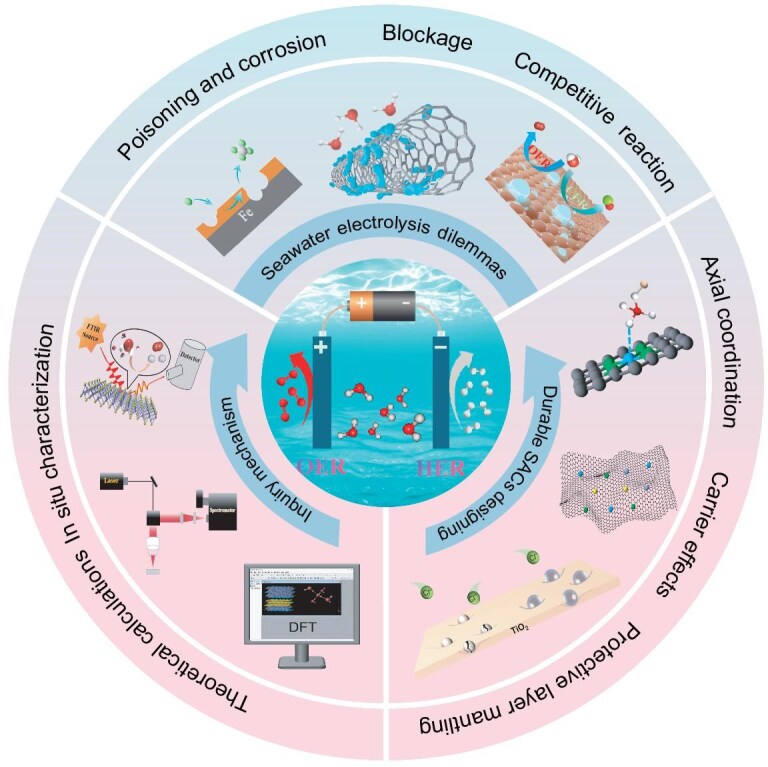
Schematic diagram of corrosion-resistant durable SACs for direct seawater cracking.

Given that seawater still needs to overcome high electric potential and corrosion reactions in a more cost-effective way, this review commences with an examination of the fundamental principles and inherent challenges associated with seawater electrolysis. Subsequently, our attention is directed toward the implementation of efficacious strategies, including axial coordination engineering, the provision of protective layer coverage and carrier effects on SACs. This was undertaken with the objective of facilitating the development of high-performance electrocatalysts that exhibit enhanced selectivity and stability in DSE (Fig. 1) [43,44]. In combination with design strategies and characterization techniques, higher selectivity and stability are presented, and the challenges and prospects for the large-scale application of SACs are considered.

## PRINCIPLES OF SEAWATER ELECTROLYSIS

The principle of seawater electrolysis is analogous to that of freshwater electrolysis, with the primary reactions occurring at the cathode as HER and at the anode as OER. In theory, a standard thermodynamic potential of 1.23 V is sufficient to drive the complete seawater electrolysis process [45]. Nevertheless, in order to guarantee the practical production of H_2_ at a sufficient rate, it is necessary to apply an additional voltage (termed an overvoltage) that will overcome the kinetic barriers and inefficiencies inherent to the cathode HER and anode OER [46]. This results in a total cell voltage that is considerably higher than 1.23 V.

In detail, HER, a two-electron transfer process, usually involves two reaction steps: the hydrogen adsorption step (Volmer step) and the desorption step of active hydrogen (Heyrovsky step and Tafel step) [47]. This can be expressed by Equations (1) and (2) for acidic and alkaline conditions, respectively:


(1) \begin{eqnarray*}
2{\mathrm{H}^+} + 2{{\mathrm{e}}^ - } \to {{\mathrm{H}}_2}.\,{E^\theta } = 0.00\,{\mathrm{V}}({\mathrm{acidic}}\,{\mathrm{conditions}})
\end{eqnarray*}

(2) \begin{eqnarray*}
&&2{{\mathrm{H}}_2}{\mathrm{O}} + 2{{\mathrm{e}}^ - } \to {{\mathrm{H}}_2} + 2{\mathrm{O}}{{\mathrm{H}}^ - }.\,{E^\theta } = - 0.83\,{\mathrm{V}}\\
&&({\mathrm{alkaline}}\,{\mathrm{conditions}})
\end{eqnarray*}

Compared to the HER, the OER is more sluggish as it involves a complex four-step transfer process, involving more procedures than the HER, as well as the desorption of reaction intermediates [48]. Therefore, OER has a higher activation potential energy barrier. Nowadays, the universally accepted mechanism includes four individual steps of electron transferring, involving three intermediates that are O*, OH* and OOH* [49]. OER is expressed as Equation (3) in acidic electrolytes and as Equation (4) in basic solutions:


(3) \begin{eqnarray*}
&&2{{\mathrm{H}}_2}{\mathrm{O}} \to 4{\mathrm{H}^+} + {{\mathrm{O}}_2} + 4{{\mathrm{e}}^ - }.\,{E^\theta } = 1.23\,{\mathrm{V}}\\
&&({\mathrm{acidic}}\,{\mathrm{conditions}})
\end{eqnarray*}

(4) \begin{eqnarray*}
&&4{\mathrm{O}}{{\mathrm{H}}^ - } \to 2{{\mathrm{H}}_2}{\mathrm{O}} + {{\mathrm{O}}_2} + 4{{\mathrm{e}}^ - }.\,{E^\theta } = 0.40\,{\mathrm{V}}\\
&&({\mathrm{alkaline}}\,{\mathrm{conditions}})
\end{eqnarray*}

Although the fundamental principles of seawater electrolysis are analogous to those of freshwater electrolysis, the intricate composition of seawater introduces a unique set of challenges that must be taken into account when considering DSE. A detailed description of this can be found in the following subsections.

Seawater is an electrolyte that lacks buffering capacity, which can result in fluctuations in pH in the vicinity of the electrode surface during electrolysis, with a potential range of 5–9 pH units [50]. This can cause the formation of salt crystals, and the deterioration of the catalyst and electrodes due to the action of other ions, microorganisms, tiny particles and bacteria, which in turn can compromise the long-term stability of the catalysts [51]. The complex chemical composition of seawater presents significant challenges to the design of electrolysis tanks. To ensure optimal performance in this challenging environment, it is necessary to upgrade and optimize a range of components, including diaphragms, electrodes, plates, auxiliary systems and more [19]. Furthermore, the competitive reaction of the OER at the anode, as represented by the CER, is an important factor to selectively facilitate the seawater splitting. Additionally, Cl^−^ can irreversibly damage electrodes through corrosion, particularly affecting anodes, and lead to a swift decline in seawater electrolysis performance [52,53]. Consequently, the investigation of anode catalysts for the selective precipitation of oxygen represents a significant challenge in the present era.

## DILEMMAS IN SEAWATER ELECTROLYSIS

### Complex and variable composition

The performance of catalysts in seawater electrolysis is profoundly influenced by the complex and variable nature of seawater composition. Factors such as temperature, pH, salinity and the presence of impurities collectively shape the activity, stability and durability of catalysts [50–53].

#### Temperature

Temperature plays a pivotal role in determining reaction kinetics, surface interactions and catalyst stability during seawater electrolysis [54]. Variations in temperature can alter the adsorption behavior of reaction intermediates and the morphology of catalytic surfaces [55]. Elevated temperatures typically decrease both the CER potential and the anodic electrode potential, which enhances chlorine gas production [53]. However, this simultaneously reduces chlorine solubility in seawater and accelerates the decomposition of hypochlorite ions, leading to diminished chlorine availability and a decline in overall electrolysis efficiency. For example, Bollu *et al*. [56] designed a CN@CNP-Pt(-)//CN@CNP(+) catalyst and evaluated its performance at different temperatures (25°C, 50°C and 75°C). At 75°C, the catalyst achieved a lower cell voltage of 1.66 V at a current density of 100 mA cm^−2^, highlighting the temperature's impact on catalytic efficiency. However, excessive temperature increases can also destabilize catalysts, emphasizing the need for thermally robust materials for seawater electrolysis.

#### pH

The pH of seawater exerts a significant influence on catalyst stability, surface charge and reaction pathways. In extreme pH conditions (highly acidic or highly alkaline), catalysts may undergo corrosion, dissolution, or structural degradation, all of which impair catalytic performance [57]. Furthermore, pH variations influence the formation of by-products such as hypochlorite or hydroxyl radicals, which can negatively affect catalyst activity and selectivity. pH also affects the reaction mechanisms, particularly the rate of proton and electron transfer, which are crucial in seawater electrolysis [50]. Each catalyst operates optimally within a specific pH range, and deviations from this range can lead to structural alterations, loss of activity, or changes in the reaction pathway [58]. Therefore, understanding and controlling pH in seawater electrolysis is critical for achieving stable and efficient catalytic performance.

#### Salinity

Seawater salinity, typically around 3.5 wt% with NaCl as the dominant salt, has a profound impact on catalyst performance [14]. High salinity introduces challenges such as Cl^−^-induced corrosion, which significantly degrades OER catalysts [59]. As the seawater is consumed during electrolysis, the concentration of Cl^−^ ions increases proportionally, further accelerating catalyst degradation [52]. Cl^−^ ions compete with water molecules and hydroxide ions for active sites on the catalyst surface, reducing the availability of these reactants and lowering catalytic activity [21]. Additionally, the structural integrity of catalysts can be compromised in high-salinity environments due to accelerated corrosion rates [16]. This is particularly problematic for catalysts with less robust materials, which may experience rapid loss of activity. Moreover, high salinity increases the conductivity of seawater, leading to greater resistance during the electrolysis process [60]. This results in elevated energy consumption, as the electric current encounters higher resistance while passing through the electrolyte [17]. Optimizing catalysts for corrosion resistance and energy efficiency under such conditions is a critical area of research for seawater electrolysis.

#### Presence of impurities

Seawater electrolysis introduces significantly more challenges compared to the electrolysis of pure water, primarily due to the diverse range of impurities present in natural seawater [22]. These impurities, including both cationic and anionic species, as well as organic microorganisms, can adversely affect the performance, stability and durability of SACs [61]. Given the unique structure of SACs, which features isolated metal atoms as active sites, addressing these challenges is critical to maintaining their functionality under harsh marine conditions [35,38].

- Cationic impurities: The most abundant cationic impurities in seawater are Ca^2+^ and Mg^2+^ ions. During seawater electrolysis, the high concentrations of these cations can lead to the formation of oxide or hydroxide deposits (e.g. Mg(OH)_2_ or CaCO_3_) on the electrode surface [57]. These deposits, commonly referred to as ‘scaling’, significantly hinder the HER by blocking active sites and disrupting hydrogen ion transfer. In SACs, such scaling can severely impact performance due to the isolated nature of their active sites, which are more susceptible to blockage [38]. Recent studies have shown that strong binding between OH^−^ and the Lewis acid layer reduces the deposition of Mg^2+^ and Ca^2+^ [62]. Developing SACs with anti-scaling properties, such as through the incorporation of hydrophobic coatings or surface modifications, is essential to counteract these effects.
- Anionic impurities: Cl^−^ are the most abundant anionic impurities in seawater and present a significant challenge due to their corrosive nature [22]. At the anode, Cl^−^ ions are readily oxidized to produce chlorine gas (Cl_2_) or hypochlorite (ClO^−^), which are highly reactive and can cause oxidative corrosion of the catalyst and associated components, such as electrode materials, bipolar plates and seals [63]. Moreover, Cl^−^ ions can directly interact with the metal active sites of SACs, forming complexes that facilitate metal dissolution and subsequent loss of catalytic activity [20]. This is particularly problematic for SACs, as the dissolution of even a single active site can lead to a measurable decline in performance. Cl^−^ may also compete with oxygen-containing intermediates for active sites, thereby reducing the OER efficiency [52]. Although Br^−^ ions are present in much lower concentrations than Cl^−^, their effects on the anode cannot be ignored due to their accumulation over time. Studies by Zhang *et al*. [64] demonstrated that Br^−^ ions cause extensive corrosion, resulting in the formation of shallow, wide pits in the protective passivation layer of the catalyst. In contrast, Cl^−^ ions create narrow, deep pits. Additionally, Br^−^ have been shown to induce exfoliation of catalyst layers on nickel-based electrodes (e.g. NiFe-layered double hydroxide (LDH)), further contributing to performance degradation. Therefore, designing SACs that are resistant to both Cl^−^ and Br^−^ corrosion is critical for seawater electrolysis applications.
- Organic and biological impurities: Seawater also contains organic microorganisms and dissolved organic matter, which can foul the catalyst surface, block active sites and reduce catalytic efficiency [65]. Such fouling can be particularly detrimental to SACs due to the atomic-scale nature of their active sites, which are more sensitive to blockages [15].

In general, the complexity of seawater composition—encompassing temperature, pH, salinity and impurities—poses significant challenges to the design and performance of catalysts for seawater electrolysis [11]. A comprehensive understanding of how these factors influence catalytic activity and stability is crucial for developing robust materials that can operate effectively in marine environments [42]. Each catalyst exhibits a specific range of optimal operating conditions, and deviations in temperature, pH or salinity can result in deactivation, structural degradation or decreased performance [22,57,59]. Future research should focus on designing catalysts that are not only resistant to these environmental variations but also capable of maintaining high efficiency and selectivity under real-world conditions.

### Marine area impacts

Global climate change and anthropogenic activities are significantly altering the marine environment, potentially exacerbating changes in seawater composition and introducing new challenges to electrocatalytic processes [66]. Rising seawater temperatures, for instance, can accelerate the kinetics of electrochemical reactions but may also destabilize catalysts and increase the formation of corrosive by-products, such as chlorine and bromine species [16]. Ocean acidification, driven by the absorption of excess atmospheric CO_2_, lowers the pH of seawater, which not only accelerates corrosion but also affects the long-term stability of catalysts [53]. Additionally, coastal areas are experiencing elevated levels of organic pollutants, heavy metals and nutrient runoff due to human activities, which can poison catalytic active sites, reduce electrolytic efficiency and necessitate advanced pretreatment processes [56]. The expansion of oxygen minimum zones (OMZs) is also altering the dissolved oxygen concentration in certain regions, requiring catalysts with enhanced responsiveness to oxygen-deficient conditions [67].

To address the regional variability in seawater composition and the impacts of global climate change, the development of catalysts that can adapt to diverse marine environments is crucial [18]. Catalyst optimization must account for the specific characteristics of seawater in different regions [16]. For example, in oxygen-rich waters, catalysts should be tailored to enhance OER selectivity, while in oxygen-depleted environments, they should emphasize improved HER activity. Furthermore, catalysts need to exhibit high tolerance to temperature and pH fluctuations to accommodate environmental changes induced by climate variability [20]. The design of impurity-resistant catalysts is particularly critical for regions with high levels of organic pollutants, heavy metals or scaling ions such as Ca^2+^ and Mg^2+^ [68]. Future advancements should also focus on improving catalyst performance while reducing production costs to ensure the economic viability of seawater electrolysis.

Looking ahead, the ongoing impacts of global climate change and human activities on the marine environment underscore the urgent need for the development of robust, adaptive catalysts with stable performance under diverse and evolving conditions [55–57]. By optimizing catalysts for specific regional seawater compositions, it is possible not only to enhance the efficiency of seawater electrolysis but also to promote the sustainable utilization of marine resources, opening new pathways for achieving low-carbon energy and environmental protection goals [60,61].

### Competitive side reactions

**Figure 2.**
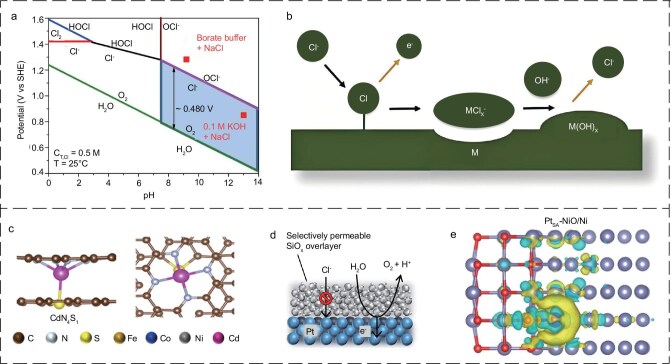
(a) Pourbaix diagram for artificial seawater model. Reproduced with permission from ref. [26]. Copyright 2024, John Wiley and Sons. (b) Schematic diagram illustrating the corrosion process of Cl^−^ on a metal surface. Reproduced with permission from ref. [74]. Copyright 2023, John Wiley and Sons. (c) The side and top view of the CdN_4_S_1_ models. Reproduced with permission from ref. [75]. Copyright 2021, John Wiley and Sons. (d) Schematic deposition of a trans-selective cover layer on the catalyst surface. Reproduced with permission from ref. [76]. Copyright 2021, American Chemical Society. (e) Computational models and localized electric field distribution of Pt_SA_-NiO/Ni. Reproduced with permission from ref. [77]. Copyright 2021, Springer Nature.

CER is an elaborate double-electron transfer process that leads to various reactions depending on the potential, pH and temperature [69]. By maintaining Cl^−^ concentration at 0.5 mol/L and the temperature at 25°C, the Pourbaix diagram for chlorination can be generated, as can be seen in Fig. 2a [26]. The formation of the molecule Cl_2_ occurs via the Heyrovsky-Volmer mechanism (Heyrovsky step: Cl* + Cl^−^ → * + Cl_2_ + e^−^; Volmer step: * + Cl^−^ → Cl* + e^−^, where * represents a catalytic active site) [70]. Thus, the strong coupling between the key intermediates of the CER and the OER often leads to their simultaneous occurrence, which is undesirable in DSE [71]. Pan *et al*. also noted that the challenges posed by competing CER, chloride corrosion and catalyst poisoning significantly hinder the development of the DSE [72].

### Poisoning and corrosion

Aggressive Cl^−^ can pierce the catalyst layers and coordinate with substrate metals, turning these metals into electrolytes and directly giving rise to the disruption of catalysts. For instance, transition-metal-based catalysts (Fe, Co, Ni and so on) are prone to dissolution in the presence of Cl^−^ due to the direct reaction with these electron-deficient metals, leading to compositional changes [73]. The corrosion mechanism of Cl^−^ on the electrode surface is illustrated in Fig. 2b [74]. Specifically, the metal surface begins to polarize in the presence of an applied electric field, thereby adsorbing Cl^−^. The adsorbed Cl^−^ then reacts with and dissolves the metal, resulting in the formation of metal hydroxides from disintegrated metal chlorides. This process consumes hydroxide ions, lowering the local pH and accelerating further metal substrate corrosion. As a consequence, Cl^−^ triggered corrosion of the metal will gradually dissolve the catalysts, directly giving rise to catalyst poisoning and poor catalytic durability. In addition, during seawater electrolysis, hydrogen is often accompanied by chlorine and halogenated elemental monomers on the counter electrode. It is also possible for active sites to be covered by highly corrosive hypochlorite by-products on the surfaces of catalysts [64].

### Blockage

The use of efficient HER has been demonstrated to offer thermodynamic advantages over OER in the case of impure cations (e.g. Mg^2+^ and Ca^2+^) due to the higher negative redox potential of these impure cations [57]. Consequently, when developing catalysts for better HER performance, the potential impact of other cation precipitates is typically overlooked, with the focus instead on enhancing activity. However, due to the lack of buffered ions in nearly neutral seawater, there is a change in the local pH near the cathode surface. In particular, HER in neutral seawater exhibits a small consumption of H_3_O^+^ or production of OH^−^ at low and high current densities, respectively. This results in an increase in pH around the cathode and an elevation of the redox potential of HER. As a result, the electrochemical reduction of impurity cations is facilitated by the reduction of the thermodynamic gap, resulting in the formation of metal oxides or hydroxides that cover the electrodes and inhibit the mass transfer of H_2_O, which in turn leads to the deterioration of HER stability. A substantial corpus of literature has documented the fact that strong binding between OH^−^ and the Lewis acid layer could alleviate sediment formation by notably reducing the capture of OH^−^ by Mg^2+^ and Ca^2+^ cations in bulk seawater electrolytes [62].

In light of the foregoing inquiries, developing corrosion-resistant catalysts through methods such as axial coordination engineering (Fig. 2c) [75], the deposition of a capping layer on the catalyst surface (Fig. 2d) serving to prevent the penetration of impurities [76], and carrier effect (Fig. 2e) is viewed as promising to mitigate side reactions and enhance the corrosion resistance and durability of catalysts in seawater [77].

## DESIGN OF CORROSION-RESISTANT AND DURABLE CATALYSTS

To date, electrocatalysts investigated for HER, OER or even overall seawater splitting have been subject to increasing levels of research. A summary of the catalytic performance and stability of different catalysts in seawater was conducted based on a review of the literature (Table 1). SACs typically demonstrate enhanced catalytic activity, attributable to their elevated surface area and synergistic effect [78]. Specifically, SACs possess a well-defined coordination configuration, which allows for the rational regulation of the metal center's coordination environment, optimizing catalytic performance [79]. Additionally, the coordination environment can be stabilized through chemical bonding with the carrier, such as through ligand bonding, which further enhances the catalyst's robustness [36]. The small size of SACs enables the selective localization of individual metal atoms at specific adsorption sites, reducing the likelihood of atom migration or agglomeration, thereby improving both catalytic selectivity and stability [38]. These factors together help explain the remarkable stability of SACs under various reaction conditions [80]. Advanced characterization techniques, such as spherical aberration corrected transmission electron microscopy (AC-TEM) and X-ray absorption fine structure (XAFS) [81], can be employed to observe and analyze the structure and properties of SACs, thereby facilitating a deeper understanding of their catalytic performance and potential for optimization [82]. However, the more complex environment of seawater has the potential to damage the efficiency of SACs. Accordingly, through the implementation of enhanced methodologies, researchers can devise and synthesize single-atom metal catalysts with tailored electronic configurations, effectively addressing the challenges posed by Cl^−^ in seawater, including catalyst deactivation, anodic chlorine oxidation side reactions and corrosion [83]. To illustrate this, a research team from Shandong University has successfully synthesized spinel NixMn_3−_*_x_*O_4_ solid solutions modified with single-atom Ir by the sol-gel method [79]. This approach has led to the development of an alkaline OER catalyst with excellent performance in electrocatalytic seawater electrolysis, exhibiting low overpotential at high current density. In addition to experimental results, the author has incorporated theoretical calculations to further assess the stability of the Ir single atom in the Ir_1_/Ni_1.6_Mn_1.4_O_4_ catalyst. The binding energy of the Ir single atom (−7.36 eV) is substantially lower than the cohesion energy (6.94 eV), suggesting that the Ir single atom exhibits thermodynamic stability. This is a strong indication that the Ir-modified catalyst not only performs effectively in terms of activity but also maintains its stability over extended periods of operation. In light of the preceding studies, we provide an analysis of the recent general design standards for electrocatalysts used in seawater splitting, with particular emphasis on the following pivotal elements: (i) axial coordination engineering, (ii) carrier effect, (iii) protective layer coverage. The aforementioned elements are indispensable for the augmentation of the electrocatalytic functionality of seawater-splitting SACs [84].

**Table 1.** OER and HER performance for the DSE of currently reported catalysts.

Catalyst	Electrolyte	Reaction	*ƞ* (mV)	*J* (mA·cm^−2^)	Stability (h)	Ref.
Fe-Co_2_P BNRs	Seawater	HER	489	10	48	[85]
WC/WP@NPC_(5.0)_	1 M KOH	HER	165	10	24	[47]
Fe_0.05_Ru_0.05_/XC-72–350°C	Seawater + 1 M KOH	HER	13	10	>24	[86]
Cu/PtNi	Alkaline seawater	HER	23	10	20	[87]
Co_4_-POM@Co-PGDY	Seawater	HER	301	10	50	[88]
NiFe-LDH/NiCo_2_O_4_/NF	1 M KOH + 0.5 M NaCl	OER	193	10	150	[18]
S-(NI,FE)OOH	Seawater + 1 M KOH	OER	300	100	100	[89]
NF/(CoMo)_0.85_Se@FeOOH	1 M KOH + 2 M NaCl	OER	276	50	75	[21]
Ni-SA/NC	Seawater + 1 M KOH	HER	139	10	14	[90]
CoNiP/Co*_x_*P	Seawater	HER	290	10	500	[91]
Na_2_Co_1−_*_x_*Fe*_x_*P_2_O_7_	0.1 M KOH + 0.5 M NaCl	OER	280	10	100	[92]
Ni-SN@C	Seawater + 1 M KOH	OER	23	10	40	[93]
NiMoFe/NM	1 M KOH + 0.5 M NaCl	OER	241	100	1500	[94]
Pt-Ni_3_S_2_/Co_9_S_8_-Sv	1 M KOH + 0.5 M NaCl	HER	183	10	24	[95]

### Axial coordination engineering

Recently, research activity in the field of electrocatalysis has significantly increased, with a particular focus on axially coordinated SACs inspired by natural enzymes [96]. The engineering of axial coordination allows monoatomic active sites to be anchored to the electrode surface. Furthermore, axially coordinated SACs act as the molecular conductor, facilitating electron transfer between the electrode and the metal monoatomic center [97]. Additionally, it modulates the electronic structure of the metal monoatomic center, thereby influencing its reactivity. In this context, axial coordination engineering demonstrates considerable potential as a novel strategy for modulating the local coordination structure and electronic structure of monoatomic sites in monoatomic catalysts. The axially coordinated monoatomic catalysts that have been reported for the electrolysis of water include atoms such as C, O, Cl and P. For example, the introduction of one or more axial ligand sites perpendicular to or non-coplanar with the M-N_4_ plane can effectively break the symmetry of the electron distribution [98]. This disrupts the electronic structure of the central monoatomic active site, optimizing the adsorption behavior and reducing the energy barrier for intermediate adsorption. Moreover, axial ligands can serve as additional adsorption sites, working synergistically with the M-N_4_ coordination to create new catalytic pathways that are more energetically favorable [96].

**Figure 3.**
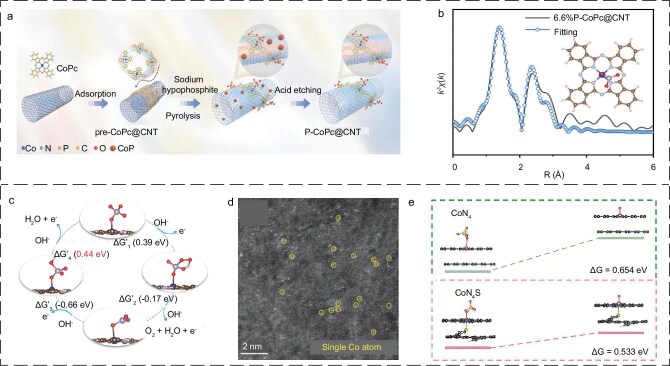
(a) Schematic illustration for the synthesis of 6.6%P-CoPc@CNT. (b) EXAFS fitting curve of 6.6%P-CoPc@CNT in R space. Inset: schematic model of 6.6%P-CoPc@CNT (C: brown, N: gray, Co: blue, P: mauve, O: red). (c) The free energy change of the OER process over PO_4_-CoPc at 1.23 V versus reversible hydrogen electrode (RHE). (a–c) Reproduced with permission from ref. [99]. Copyright 2022, John Wiley and Sons. (d) AC-HAADF-STEM image of CoN_4_S-CB. (e) Free energy changes for Co(IV)=O generation on CoN_4_ and CoN_4_S sites. (d, e) Reproduced with permission from ref. [100]. Copyright 2024, Elsevier.

By modulating Co_1_N_4_ with axial coordination, the Mao team [99] introduced active axial PO_4_^3−^ coordination sites as reaction sites to participate in the reaction, which directly lowered the energy barrier by changing the reaction path and thus improved the OER activity of the catalyst (Fig. 3a–c). This study sets out a strategy for introducing new active sites by axial coordination engineering of SACs, with the aim of modifying the reaction path and thus enhancing the catalyst activity. A single-atom cobalt catalyst (CoN_4_S-CB) with axial S coordination was developed by Zhu *et al*. [100] using an axial coordination strategy. The catalyst activates peroxymonosulfate (PMS) to generate high-valence Co-Oxo species (Co(IV)=O) with high selectivity, and effectively degrades sulfamethoxazole (SMX) in all complex environments via the oxygen atom transfer (OAT) reaction. Based on the Fourier transform extended X-ray absorption fine structure (FT-EXAFS) spectra in R-space fitting results, the atomic configuration model of CoN_4_S-CB was visualized as a cytochrome P450 monooxygenase-like single-atom nanozyme with an axial S ligand and four horizontal N ligands (Fig. 3d). Density functional theory (DFT) calculations indicate that axial S coordination modifies the 3d orbital electron distribution of Co atoms, reducing the PMS desorption energy barrier and the Co(IV)=O generation free energy (Δ*G*) (Fig. 3e). This work elucidates the potential mechanism of axial S-liganded single-atom cobalt catalysts for controlling the PMS activation pathway, thereby providing theoretical guidance for future developments in catalyst design for PMS activation and high-valence metal-oxide-mediated water purification. This strategy has been less frequently employed in the context of SACs and even less so in the case of seawater. Consequently, there is considerable scope for further research in this area.

It is worth noting, however, that the current studies on SACs based on axial coordination design for seawater electrolysis remain relatively limited [96]. While challenges such as unwanted side reactions (e.g. Cl_2_ production in some cases) suggest that axial coordination engineering is not universally applicable, these findings also highlight the need for further research in this field [79]. Axial coordination SACs offer substantial potential to address these challenges and provide new perspectives on single-atom catalysis mechanisms [98]. We believe that this approach holds significant promise for advancing the design and performance of SACs in seawater electrolysis.

### Carrier effects

In contrast to axial coordination engineering, loaded SACs are widely used and have been studied more extensively. Exploiting metal–carrier interactions through structural design as well as exploiting synergistic promotion is among the essential strategies for enhancing catalytic efficiency. Single atoms are ideal models for the electronic metal–support interaction due to their unique electronic structure and maximum atom utilization [101,102]. Loaded SACs can form a single-atom dispersion state on the carrier. This is connected with the carrier through chemical bonding and has a relatively stable structure, with excellent activity, selectivity and stability [103]. The carriers usually include metal oxides/hydroxides, nitrides, carbon materials and so on.

#### Metal oxides/hydroxides

Metal oxide/hydroxide-loaded SACs have been the focus of considerable attention among the many carriers, due to the properties that they exhibit at the surface in terms of pH and redox. Metal oxide/hydroxide carriers not only facilitate the dispersion and stabilization of metal atoms, but also interact with them, frequently giving rise to surface charge transfer, metal structure modification and modulation of molecular adsorption [104,105]. These interactions ultimately influence the performance of the catalyst.

**Figure 4.**
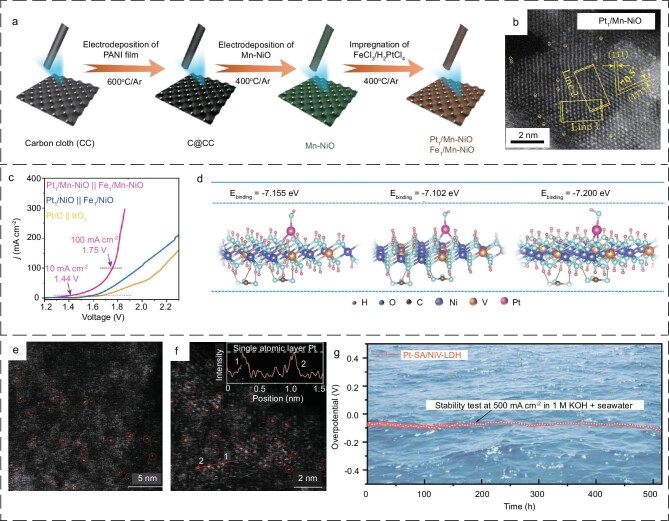
(a) Schematic diagram of the synthesis procedure for Pt_1_/Mn-NiO and Fe_1_/Mn-NiO. (b) HAADF-STEM image of Pt_1_/Mn-NiO. (c) The linear sweep voltammetry (LSV) curves of Pt_1_/Mn-NiO||Fe_1_/Mn-NiO for overall water splitting. (a–c) Reproduced with permission from ref. [106]. Copyright 2023, American Chemical Society. (d) Pt single atoms supported on the Ni top, V top and Ni-V hollow sites of NiV-LDH. (e, f) AC-STEM micrographs of Pt-SA/NiV-LDH. (g) The stability test of Pt-SA/NiV-LDH. (d–g) Reproduced with permission from ref. [107]. Copyright 2024, John Wiley and Sons.

Chen *et al*. [106] put forward a methodology for the modulation of anchored metal single atoms, which involved the manipulation of the surface polarization of the carrier. This approach was shown to be effective in the fabrication of electrocatalysts that were capable of functioning under alkaline seawater conditions. In particular, the authors introduced Mn doping into the weakly polarized NiO nanosheets to modulate their surface polarization, which resulted in the modulation of the electronic interactions between the anchored Pt/Fe single atoms and the NiO carrier. Figure 4a illustrates the synthesis procedure of Mn-NiO nanosheets designed using Pt/Fe single atoms, which encompasses both electrodeposition and impregnation steps. The transmission electron microscopy (TEM) image (Fig. 4b) shows the presence of atomically dispersed platinum and iron atoms in Pt_1_/Mn-NiO and Fe_1_/Mn-NiO, which suggests that they are characteristic of SACs. The catalyst displays excellent alkaline seawater decomposition performance, requiring only 1.44 V to achieve 10 mA cm^−2^ (Fig. 4c). It is anticipated that the findings of this research will offer novel insights into the advancement of clean energy and the pursuit of more sustainable applications of electrocatalytic holistic seawater decomposition technology. Wang *et al*. [107] immobilised Pt single atoms on self-supported nickel-vanadium LDH (NiV-LDH), resulting in the production of highly efficient and stable self-supported monoatomic Pt-SA/NiV-LDH catalysts for seawater electrolysis for hydrogen production. The authors initially conducted a DFT calculation to analyze the binding energies of Pt atoms in different positions on NiV-LDH carriers. They then employed a screening and identification process based on the principle of lowest binding energy to ascertain more stable atomic configurations (Fig. 4d). Subsequently, the Pt atoms were dispersed uniformly on ultrathin NiV-LDH nanosheets through an ethylene-glycol-assisted hydrothermal method. This resulted in the formation of a self-supporting Pt-SA/NiV-LDH atomic catalyst. The aberration-corrected scanning transmission electron microscope images revealed the presence of a considerable number of Pt atoms distributed homogeneously on the NiV-LDH carrier (Fig. 4e and f). Furthermore, the catalyst demonstrated efficacy in hydrogen production via seawater electrolysis under alkaline conditions at 500 mA cm^−2^ for a duration exceeding 500 hours (Fig. 4g).

#### Nitrides

Nitrides, as monoatomic carriers, exhibit excellent catalytic activity, which effectively promotes the coordination of the active center atoms of monoatomic catalysts, thereby enhancing their adsorption and activation of reaction substrates [108]. Secondly, the transfer of electrons between the active center atoms and the nitrides in SACs results in the partial charging of the active center atoms [109]. This, in turn, affects the adsorption state of the reaction substrate, thereby achieving a highly selective catalytic reaction. Furthermore, the high stability of nitrides puts them at a significant advantage as single-atom carriers, ensuring that SACs maintain stable catalytic performance over extended periods of time.

Lee *et al*. [110] designed a bifunctional electrocatalyst for full pH water decomposition, comprising a single-atom Co-modified molybdenum disulfide nanosheet assembled on arrays of metal nitride nanorods. In this study, interactions between Co SACs and MoS_2_ led to the modulation of the electronic structure, thereby optimizing the intermediate adsorption/desorption capacity. The unique 3D nitride structure, which provided a larger surface area and ample active sites, resulted in an enlarged contact surface between the reactants and the active sites, facilitating permeation/diffusion of the reactants and release of the gaseous species.

**Figure 5.**
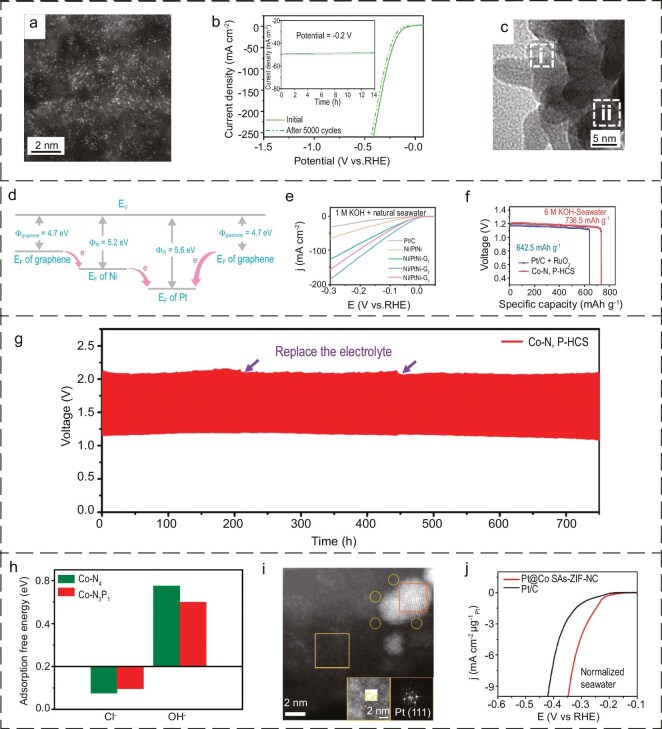
(a) Isolated Ni atoms are distributed across the nitrogen-doped carbon support. (b) LSV curves of Ni-SA/NC before and after 5000 cyclic voltammetry cycles. (a, b) Reproduced with permission from ref. [90]. Copyright 2020, John Wiley and Sons. (c) High magnification TEM image of Ni/PtNi-G_2_. (d) A schematic illustration of the synergistic electron donation from graphene and Ni to Pt in Ni/PtNi-G. (e) HER polarization curves of different catalysts in alkaline natural seawater. (c–e) Reproduced with permission from ref. [120]. Copyright 2021, the Royal Society of Chemistry. (f) Specific capacities normalized to the mass of consumed Zn in batteries at a current density of 10 mA cm^−2^ for S-ZABs driven by Co−N,P−HCS compared with commercial Pt/C+RuO_2_. (g) Galvanostatic discharge–charge cycling curves of the Co−N,P−HCS-based seawater Zn−air batteries. (h) Calculated adsorption-free energies of Cl^−^ and OH^−^ on the symmetric Co−N_4_ and asymmetric Co−N_3_P_1_ models, respectively. (f–h) Reproduced with permission from ref. [121]. Copyright 2022, John Wiley and Sons. (i) HAADF-STEM image of Pt@Co SAs-ZIF-NC. (j) The LSV curves of different samples in seawater. (i, j) Reproduced with permission from ref. [122]. Copyright 2021, Elsevier.

Mxenes, a nano-lamellar family of ternary carbides/nitrides, have been widely studied as a highly secure structural material [111]. Nevertheless, the direct application of Mxenes in electrocatalysis has yet to be realized. Based on this, Huang *et al*. [112] reported single-atom-thick A layers in nano-laminated M_n+1_AX_n_ phases. It would appear that the application of SACs in seawater has yet to be fully realized. The M-N_4_ site is the most frequently reported active site. In a recent study, Kou and colleagues [90] reported a simple surfactant-assisted method for the synthesis of Ni-N_3_ and Ni-N_4_ coordinated Ni single-atom electrocatalysts (Ni-SA/NC) and conducted a comprehensive investigation into their catalytic performance in alkaline seawater electrolyte. High-angle annular dark field scanning transmission electron microscopy (HAADF-STEM) revealed that the nickel single atoms were homogeneously distributed across the surface and surrounded the micropores and mesopores of the nitrogen-doped carbon carriers (Fig. 5a). In addition, the catalyst demonstrated excellent stability in alkaline seawater over 5000 cycles, with no notable decline in activity after 14 hours of continuous electrolysis. The well-isolated dispersion of individual Ni atoms anchored in the porous N-doped carbon results in a considerable number of active sites with tunable coordination environments, which demonstrates considerable potential for the development of inexpensive and efficient SACs for seawater decomposition (Fig. 5b).

Nitrides, when employed as carriers for SACs, provide a catalytic environment of optimal efficiency, selectivity and stability for SACs, due to the distinctive physical and chemical attributes of nitrides. Additionally, the electron transport facilitated by nitrides enhances the overall performance of SACs.

#### Carbon-based materials

Carbon-based materials represent a novel type of SAC carrier, exhibiting exceptional electrical conductivity, and thermal and chemical stability [113]. The dispersion and activity of SACs can be controlled by adjusting the surface functional groups of carbon-based carriers, thereby enhancing the efficiency and selectivity of catalysts [114]. Among the carbon-based carriers, graphene and carbon nanotubes are two of the most extensively researched [115,116]. They possess a large specific surface area and superior electronic transfer performance, which affords them a wide range of potential applications in the preparation and utilization of catalysts [116].

The combination of graphene-loaded monoatomic electrocatalysts represents a promising avenue for enhancing the efficiency of seawater electrolysis [117]. This approach leverages the high conductivity and stability of graphene with the high activity of monoatomic catalysts, offering a potential solution for improving the process efficiency of seawater electrolysis [118,119]. Yang's team [120] prepared Ni/PtNi heterojunction composite electrocatalysts loaded on graphene via a single-step hydrothermal process (Fig. 5c). In this study, a directional electron migration pathway was constructed from graphene to Ni and finally to Pt, comprising a stepped work-function gradient with Ni (5.2 eV), which has a moderate work function, as a bridge for the transport of electrons. Additionally, the d-π heterostructure between the metal Pt and graphene was enhanced, and the electron transport efficiency was improved, resulting in the construction of an electron-rich surface for the Pt-based material (Fig. 5d). Consequently, the catalyst demonstrated effective resistance to corrosion by negatively charged Cl^−^ ions and exhibited high catalytic activity in alkaline seawater (Fig. 5e). This work offers insights and theoretical guidance on the interaction between carbon material carriers and metal catalysts in electrocatalysts.

The process of carbon-nanotube-loaded monoatomic electrocatalysts represents a method of efficiently catalyzing the electrolysis of seawater, whereby the special physical and chemical properties of carbon nanotubes are exploited. In this process, carbon nanotubes are employed to facilitate the transportation or stabilization of monoatomic metal catalysts, thereby enhancing the efficiency of electrolysis while mitigating the corrosion of ions in seawater. Huang *et al*. [121] employed DFT calculations to design an atomically asymmetric Co-N_3_P_1_ structure and then implanted hollow carbon spheres, which exhibited excellent oxidation-reduction reaction/OER/HER performance in seawater and were observed to work for 1000 h continuously in seawater electrolysis (Fig. 5f–h). This presents a promising avenue for the development of corrosion-resistant multifunctional catalysts in seawater-based electrolytes, offering high efficiency and resilience to Cl^−^ ions.

Porous carbon-matrix-derived metal-organic frameworks are also one of the most commonly used carriers. Mu *et al*. [122] successfully prepared Pt@Co SACs-zeolite-based imidazole framework-N-doped carbon materials catalysts by capitalizing on the segregation of Pt by cobalt monoatomic sites, the robust interactions between Co single atoms and Pt, and the confinement of metal-organic frameworks derived from porous carbon substrates. HAADF-STEM revealed that Co predominantly exists as single atoms in proximity to Pt nanoparticles, and the hydrogen precipitation performance matches with Pt/C in alkaline seawater. Furthermore, the hydrogen precipitation performance in alkaline seawater was found to be similar to Pt/C (Fig. 5i and j).

There is a wide variety of SAC carriers, each of which has its unique advantages. With the continuous development of catalytic chemistry, the research and application of SACs in seawater will become more and more extensive in the future.

### Protective layer mantling

It is an effective strategy to form a protective layer on the SACs surface to prevent corrosion of active sites or substrate materials by Cl^−^ while maintaining high catalyst activity [123,124]. A substantial corpus of empirical evidence attests to the efficacy and reliability of this methodology when applied to electrolytic seawater, thus substantiating its viability.

**Figure 6.**
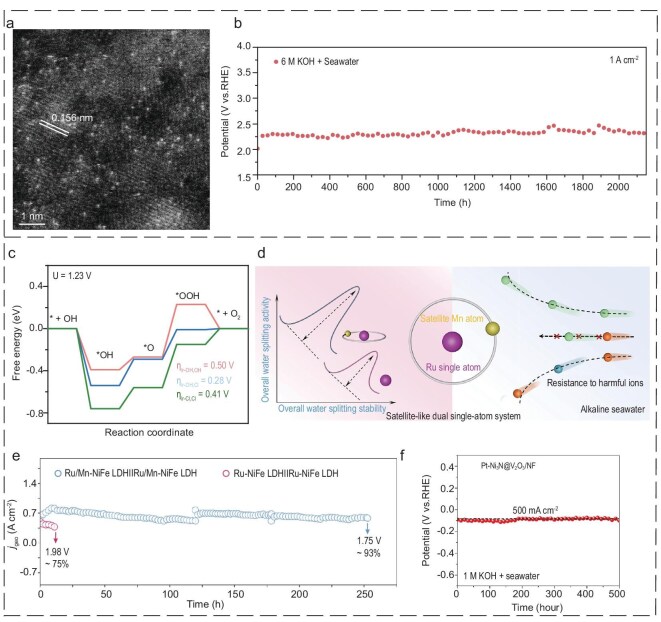
(a) HAADF-STEM image of Ir/CoFe-LDH. (b) The stability test in real seawater. (c) The activation energy of Ir/CoFe-LDH with three coordination states. (a–c) Reproduced with permission from ref. [60]. Copyright 2024, Springer Nature. (d) Schematic diagram for satellite-like shielding for dual single-atom catalysis in electrolysis alkaline seawater. (e) Chronoamperometry profiles of catalysts. (d, e) Reproduced with permission from ref. [127]. Copyright 2024, Elsevier. (f) Chronopotentiometry curves of Pt-Ni@NiMoN/NF working at 500 mA cm^−2^ for 500 hours in 1 M KOH + seawater. Reproduced with permission from ref. [55]. Copyright 2024, The American for the Advancement of Science.

The detrimental effects of severe catalyst structural damage and deactivation can be mitigated through the leaching of Cl^−^ from the catalyst lattice and the prevention of Cl^−^ invasion from the electrolyte [125]. This approach can facilitate the attainment of a certain degree of dynamic equilibrium. By employing structural buffering strategies, other metal oxides may be anchored to suitable anions, thereby enhancing catalytic performance in seawater [126]. Sun *et al*. [60] designed an atomic-scale Ir catalyst for electrochemical seawater oxidation on Co-Fe LDH (Ir/CoFe-LDH). The HAADF-STEM image clearly demonstrates the presence of bright spots, which can be attributed to individual Ir atoms anchored on the CoFe-LDH surface (Fig. 6a). Additionally, the catalyst demonstrated stable operation in real seawater up to 10 mA cm^−2^ with 208 mV and maintained stable operation for over 2000 hours at 1 A cm^−2^ (Fig. 6b). In contrast to conventional catalysts for seawater electrolysis, which entirely exclude Cl^−^ adsorption, the Ir atomic sites on CoFe-LDH permit Cl^−^ adsorption, thereby modulating the electronic structure of the Ir active center (Fig. 6c). This work exploits the abundant Cl^−^ ions present in seawater, with the objective of dynamically regulating the coordination environment of iridium single atoms loaded on CoFe-LDHs. This dynamic regulation has the effect of lowering the energy barrier of the rate-determining step in the OER process, thereby improving the comprehensive performance of the OER in simulated alkaline seawater.

A Lewis acid layer was introduced over a range of common catalysts with the objective of manipulating the local reaction microenvironment of their DSE. This strategy may be applied to a range of catalysts without the need for a specially designed catalyst or electrolyzer [54]. Zhao's group [127], inspired by the protective effect of the Moon on the Earth, has developed a groundbreaking satellite-based shielding tactic for bis-monoatomic catalysis. The protection is attributed to the presence of the monoatomic Mn from the satellite, which introduces a substantial number of robust Lewis acid sites. These sites are tightly combined with the OH^−^ generated at the cathode *in situ*, thereby preventing an increase in pH in the surrounding seawater and thus restraining metal deposition (Fig. 6d). Furthermore, the rapid replenishment of OH^−^ results in the formation of a negative charge layer at the anode, thus resisting Cl^−^ attack, which creates an alkaline freshwater-like microenvironment in the vicinity of the electrode. Consequently, the Ru/Mn-NiFe-LDH displays remarkable catalytic capabilities in seawater under alkaline conditions, attaining 1.66 V, with 2000 mA cm^−2^ at 6 M KOH and 80°C, and an exceptional durability of >252 hours at industrial-grade current intensity (Fig. 6e). This work provides unique insights and innovative strategies for the novel construction of excellent efficient dual single-atom catalysts for comprehensive alkaline seawater decomposition at industrial-grade current densities. Hu *et al*. [55] introduced a Pt-Ni_3_N@V_2_O_3_/NF electrocatalyst featuring a dual active-site structure. The addition of a V_2_O_3_ protective layer has been demonstrated to enhance the catalytic activity of the material in question, via a number of mechanisms, including increased interfacial conductivity, improved water adsorption and enhanced hydrogen desorption. Additionally, the V_2_O_3_ protective layer modulates the local reaction environment at the catalytic site in accordance with the Lewis acid properties. The excess hydroxide captured by the V_2_O_3_ layer effectively repelled negatively charged Cl^−^, consequently resulting in a reduction in the extent of Cl^−^ attack on the active site. In addition, anion exchange membrane electrolysis cells exhibited excellent activity and durability under harsh industrial conditions (Fig. 6f).

Currently, the exposed surface area of an electrocatalyst is closely related to catalytic performance. To avoid corrosion/toxicity of the active sites, the main strategy is to adjust the surface structure and electronic state of the material to expose more active sites [128]. In the case of SACs, research has concentrated on modulation techniques, including axial coordination engineering, carrier synergies and protective layer design, with the objective of enhancing the intrinsic activity and stability of the catalysts.

## MECHANISM EXPLORATION

### Theoretical calculations

Theoretical calculations, particularly DFT, provide comprehensive electronic structure data and reaction pathway analysis, thus enabling the prediction of catalyst performance and the optimization of its design [129].

- Theoretical calculations can evaluate the corrosion resistance of catalysts in seawater by analyzing suspended bonds, surface energy and electrode reaction kinetic parameters [63]. For instance, the strength and characteristics of suspended bonds directly influence the adsorption capacity and catalytic activity of the catalyst [129]. Weak suspended bonds may result in poor adsorption of intermediates, while overly strong bonds may lead to excessive adsorption, causing catalyst poisoning or blocking key active sites. Surface energy calculations are essential, as materials with high surface energy tend to be more reactive and susceptible to environmental corrosion [121]. Thus, catalysts with optimized surface energy can balance activity and stability, reducing their vulnerability in seawater electrolysis conditions. By integrating these parameters, it becomes possible to identify catalysts with optimal bond strength, low surface energy and favorable reaction kinetics, ensuring enhanced corrosion resistance and long-term stability during seawater electrolysis [54].
- Calculating the adsorption energy (*E*_ads_) of various ions (e.g. Cl^−^ and SO_4_^2−^) provides critical insight into the impact of seawater components on catalyst surfaces [130]. For instance, a high *E*_ads_ for Cl^−^ ions on the catalyst surface indicates a strong adsorption capacity, which may accelerate corrosion and reduce stability. Conversely, low *E*_ads_ values for Cl^−^ adsorption suggest that the catalyst resists Cl^−^-induced degradation, enhancing its long-term stability [121]. Additionally, theoretical calculations of *E*_ads_ can help identify catalysts that preferentially adsorb O_2_ and water molecules over corrosive ions, thereby improving catalytic performance and corrosion resistance in seawater environments [130].
- The stability of catalysts in seawater environments can also be assessed using thermodynamic calculations [60]. The enthalpy change (Δ*H*) and free energy change (Δ*G*) of key reactions provide insight into the feasibility and stability of the catalyst under operational conditions [120]. A consistently negative Δ*G* value across various reaction pathways indicates thermodynamic stability, while small Δ*H* values suggest minimal energy input is required, enhancing operational efficiency [100]. By comparing the Δ*G* values for competing pathways (e.g. OER vs. CER), researchers can determine the selectivity and long-term stability of catalysts under different seawater compositions and environmental changes.
- Molecular dynamics (MD) simulations enable the modeling of catalyst behavior under realistic operating conditions, incorporating factors such as temperature, pressure and reactant concentration [131]. By observing molecular motion and interactions over time, MD simulations reveal the ageing mechanisms of catalysts, including structural degradation, ion adsorption behavior and changes in active site geometry [68]. These simulations provide targeted suggestions for improving catalyst designs to mitigate long-term performance decay and corrosion under seawater conditions.

**Figure 7.**
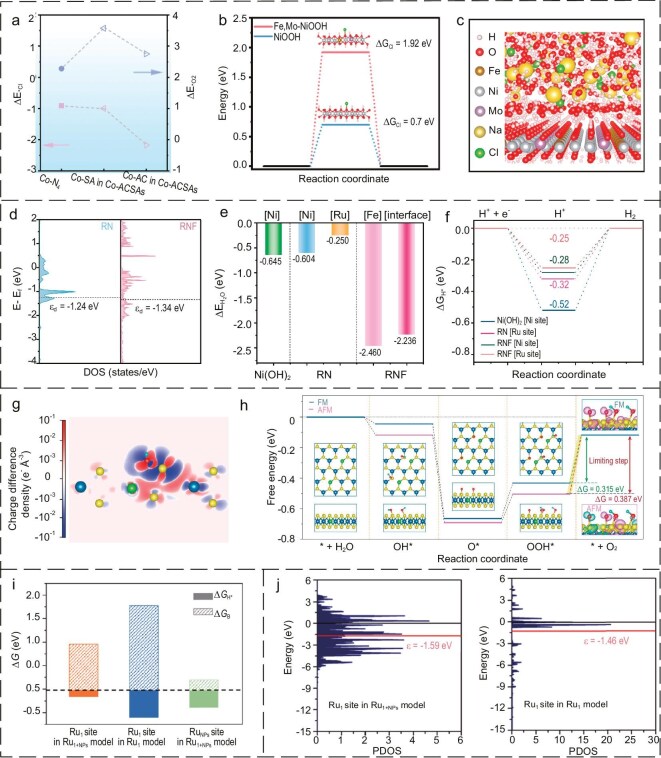
(a) Calculated adsorption energy of Cl^−^ and O_2_ molecules to different active sites in Co-ACSAs and Co single atoms. Reproduced with permission from ref. [132]. Copyright 2024, Elsevier. (b) Gibbs free energy of chlorine adsorption on O atoms at different site surfaces of the Fe,Mo-NiOOH and NiOOH. (c) A partial image of the equilibrium configuration of the catalyst-electrolyte system. (b, c) Reproduced with permission from ref. [131]. Copyright 2024, Elsevier. (d) The *ε*_d_ of RuSAs/Ni(OH)_2_ (RN) and RuSAs/Ni(OH)_2_@FeOOH (RNF). (e) Diagram of *G*_H_2_O_ of RN and RNF. (f) Diagram of *G*_H*_ of RN and RNF. (d–f) Reproduced with permission from ref. [133]. Copyright 2024, Elsevier. (g) The charge difference density a single Ni_Mo_ site upon the adsorption of *OH. (h) Free energy (*G*) profile of the OER with the corresponding adsorption configurations of reaction intermediates over ferromagnetic (FM) and antiferromagnetic (AFM) Ni_1_/MoS_2_. (g, h) Reproduced with permission from ref. [134]. Copyright 2023, Springer Nature. (i) Relationship between the computed Δ*G*_H*_ and Δ*G*_B_ values for the Ru sites of Ru_1+NPs_ and Ru_1_ models. (j) The d-projected DOS for the Ru_1_ site of Ru_1+NPs_ and Ru_1_ models. (i, j) Reproduced with permission from ref. [135]. Copyright 2022, American Chemical Society.

For example, Deng *et al*. [132] designed a catalyst model comprising Co-atom clusters (Co-ACs) surrounded by satellite Co single atoms (Co-SAs). Computational modeling showed that Co-ACs exhibited strong adsorption for Cl^−^, while Co-SAs displayed a weaker Cl^−^ adsorption capacity but a stronger O_2_ adsorption capacity (Fig. 7a). Guided by these theoretical insights, the Co-AC and Co-SA structures were stabilized on N-doped carbon substrates using a high-temperature flash burnout (HTS) strategy. The resulting catalyst achieved a remarkable stability of at least 380 hours at a current density of 5 mA cm^−2^, demonstrating the utility of theoretical modeling in catalyst design. Additionally, Meng *et al*. [131] utilized theoretical analysis and MD simulations to demonstrate that a negatively charged catalyst surface repels Cl^−^ ions and attracts H_2_O molecules. This process facilitates the formation of a protective water film, significantly enhancing the corrosion resistance and stability of the Fe, Mo-NiOOH catalyst (Fig. 7b and c). These insights were validated experimentally, showing improved stability in seawater electrolysis applications.

Liu *et al*. [133] synthesized a Ru_SAs_-doped Ni(OH)_2_ coupled with FeOOH clusters to form a porous nanowire hetero-structured catalyst, termed RNF. Theoretical calculations showed excellent bifunctional electrocatalytic performance in agreement with experimental results, as the synergistic interaction of Ru_SAs_-doped Ni(OH)_2_ with FeOOH not only enhanced the charge transfer capacity (Fig. 7d–f) but also accelerated the dissociation of water and optimized the adsorption intermediates in electrochemical processes. The combination of heterogeneous single atomic spin catalysts (SASCs) with magnetic fields represents a highly effective method for accelerating chemical reactions, enhancing metal utilization and improving reaction efficiency. Lu *et al*. [134] used a scalable hydrothermal method to prepare a variety of single-atom spin catalysts (SASCs). Among them, Ni_1_/MoS_2_ was superior in stability to IrO_2_ as an OER catalyst in both pure water and seawater splitting batteries, showing its great potential in practical seawater splitting technology. Theoretical calculations revealed the high performance of Ni_1_/MoS_2_ for the magnetic-field-enhanced OER, attributed to the magnetic-field-induced spin alignment and spin density optimization from the field-controlled S(p)-Ni(d) hybridization, optimizing the adsorption energy of the radical intermediate during the reaction to reduce the overall reaction potential barrier (Fig. 7g and h). Furthermore, Pan *et al*. [135] developed an efficient catalytic material (Ru_1_ + NPs/N-C), comprising monoatomic Ru-N_4_ sites and Ru nanoparticles immobilized on nitrogen-doped carbon via a coordination pyrolysis strategy utilizing melamine-formaldehyde resins. This material exhibited seawater electrolytic activity and stability. DFT calculations demonstrated that Ru nanoparticles facilitate the dissociation of water, reducing the free energy required at the Ru-N_4_ site (Fig. 7i and j). The Ru nanoparticles enable the dissociation of H_2_O into adsorbed hydrogen, promoting the formation of H_2_ from the monoatomic Ru-N_4_ site and its subsequent desorption. This work offers a novel approach to the design of electrocatalysts for the efficient production of hydrogen by large-scale seawater electrolysis.

Theoretical calculations have proven invaluable in advancing the understanding of SAC stability and corrosion resistance [131]. By predicting and optimizing parameters such as bond strength, surface energy, adsorption energies and thermodynamic stability, researchers can design more robust and efficient catalysts for seawater electrolysis [129]. Future efforts will likely focus on integrating these theoretical approaches with experimental validation to further refine catalyst performance.

### *In situ* characterization

**Figure 8.**
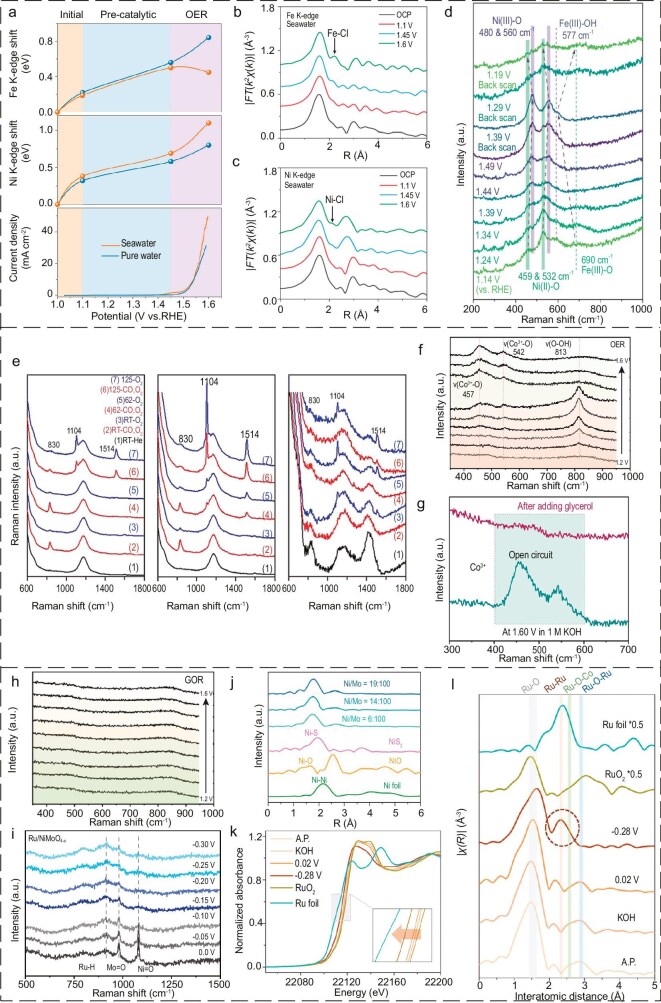
(a) Shifts in the Fe and Ni K-edge position extracted from *in-situ* X-ray absorption near edge structure (XANES) spectra and corresponding OER currents of NiFe-LDH. (b) Fe K-edge and (c) Ni K-edge in alkaline seawater. (a–c) Reproduced with permission from ref. [140]. Copyright 2023, John Wiley and Sons. (d) Operando EC-Raman spectra of (Ni,Fe)O(OH)@NiCoS NAs in the 1.0 M KOH + 0.5 M NaCl electrolyte. Reproduced with permission from ref. [141]. Copyright 2025, Elsevier. (e) *In-situ* Raman spectra of 0.2 Pd/CeO_2_, 0.4 Pd/CeO_2_ and 4 Pd/CeO_2_. Reproduced with permission from ref. [144]. Copyright 2021, John Wiley and Sons. (f) *In-situ* Raman spectra of a-Ru-CoP_2_ for OER. (g) Raman spectra of a-Ru-CoP_2_ at 1.6 V (vs. RHE) after OER and the addition of glycerol. (h) *In-situ* Raman spectra of a-Ru-CoP_2_ for GOR. (f–h) Reproduced with permission from ref. [142]. Copyright 2024, Elsevier. (i) *In-situ* Raman spectra of Ru/NiMoO_4−_*_x_*. Reproduced with permission from ref. [139]. Copyright 2023, John Wiley and Sons. (j) Ni K-edge Fourier-transformed EXAFS spectra of Ni_1_/MoS_2_ SASCs with different Ni contents. Reproduced with permission from ref. [134]. Copyright 2023, Springer Nature. (k) *In-situ* Ru K-edge XANES spectra and (l) the corresponding EXAFS R-space spectra for the Ru/NC at various potentials for HER and references. Reproduced with permission from ref. [147]. Copyright 2023, John Wiley and Sons.

In recent years, the production of hydrogen from electrolytic seawater has attracted considerable interest as a sustainable method of hydrogen production [136]. SACs have emerged as a prominent area of research due to their efficient performance and excellent selectivity [34]. The performance of SACs is closely related to the characteristics of their active sites, electronic structures and reaction mechanisms [137]. Therefore, a comprehensive understanding of the effects of these factors on catalytic performance is essential. *In-situ* characterization techniques play a vital role in understanding and optimizing the activity, stability and durability of electrocatalysts, particularly for DSE used in seawater electrolysis [138]. DSEs face unique challenges due to the corrosive nature of seawater, which contains a variety of ions (e.g. Cl^−^) and fluctuating environmental conditions [64,74]. Here, we explore specific applications of *in-situ* techniques in addressing these challenges:

- Monitoring environmental fluctuations (temperature, pH) and their effects on DSE performance: Seawater electrolysis operates under varying environmental conditions where factors such as temperature and pH directly impact the thermodynamics and kinetics of the reaction [54,57]. *In-situ* techniques, such as *in-situ* Raman spectroscopy or electrochemical impedance spectroscopy (EIS), enable real-time monitoring of these parameters, providing dynamic data on reaction behavior [138]. For DSE, this is critical in optimizing experimental conditions to maintain stable operation and minimize structural degradation caused by environmental variability. Wang *et al*. [139] investigated hydrogen spillover mechanisms in Ru/NiMoO_4−_*_x_* catalysts enriched with oxygen vacancies. Using *in-situ* Raman spectroscopy, they tracked H^∗^ adsorption dynamics and correlated this with oxygen-vacancy-mediated electronic effects. Their results revealed a direct link between spillover enhancement and HER activity, providing insights for the development of HER-specific DSE materials.
- Evaluating catalyst stability against seawater ions and contaminants: The presence of aggressive ions, such as Cl^−^, SO_4_^2−^ and Mg^2+^, can lead to catalyst degradation and loss of stability [15,57,62]. *In-situ* characterization methods allow researchers to analyze the performance of DSE under realistic seawater compositions. For instance, *in-situ* X-ray absorption spectroscopy (XAS) can track the oxidation states of active sites in the presence of chloride ions, revealing corrosion dynamics [81]. Real-time monitoring enables the design of DSE coatings that resist ion-induced degradation and selective adsorption of intermediates, ensuring long-term operational stability. Qiao *et al*. [140] employed *in-situ* XAFS to show that Cl^−^ adsorption on Fe sites in catalysts inhibited Fe dissolution while promoting the formation of OER-active Ni sites (Fig. 8a–c). This dual effect improved both stability and activity, providing critical insights for the design of durable DSE coatings.
- Identifying side reactions and optimizing reaction pathways for DSE: One major challenge in seawater electrolysis is the competition between the OER and the CER [20]. *In-situ* techniques help identify the occurrence of side reactions and their impact on the kinetics of the main reaction [63]. For example, *in-situ* Raman spectroscopy and operando electrochemical-Raman (EC-Raman) techniques can capture real-time changes in catalyst surfaces, enabling researchers to tune experimental parameters to suppress CER and enhance OER selectivity [141]. This is particularly relevant for DSE, where the suppression of side reactions directly enhances the efficiency and selectivity of the electrolysis process. Na *et al*. [141] used operando EC-Raman to study hydroxyl intermediate stabilization during OER in seawater (Fig. 8d). Their findings demonstrated that stabilizing these intermediates effectively suppressed Cl^−^-induced corrosion, enhancing catalyst stability and activity in seawater environments.
- Real-time observation of catalyst reconfiguration and degradation: During long-term seawater electrolysis, DSE materials often undergo structural and compositional changes, which can affect their activity and stability [57,65]. *In-situ* characterization techniques facilitate real-time monitoring of these changes. *In-situ* XAS and XAFS analysis can detect the reconfiguration of active sites and the formation of new phases, providing insight into the degradation mechanisms of DSE [81]. This allows researchers to develop more robust catalysts and coatings tailored to withstand the harsh marine environment. Peng *et al*. [142] demonstrated the application of *in-situ* Raman spectroscopy to study Ru-doped CoP_2_ (Ru-CoP_2_) catalysts for seawater electrolysis coupled with glycerol oxidation. They observed surface structural changes during OER, identifying CoOOH species at specific Raman peaks (457 and 542 cm^−2^). The disappearance of these peaks during the introduction of glycerol indicated selective catalytic behavior. These findings highlight the importance of surface restructuring for improved selectivity and stability in seawater applications.

*In-situ* characterization offers unparalleled insights into the dynamic processes occurring during seawater electrolysis, particularly for DSE [138]. By enabling real-time observation of environmental impacts, side reactions, catalyst reconfiguration and ion-induced degradation, these techniques provide a deeper understanding of the structure-stability-activity relationship in seawater electrolysis [136,138]. Moving forward, the integration of advanced *in-situ* techniques with theoretical modeling and experimental validation will be critical for the development of next-generation DSE materials tailored to harsh marine environments.

Advances in *in-situ* characterization techniques have enabled the real-time monitoring of catalyst surface reactions, allowing the observation of the dynamic changes occurring in the catalysts under actual reaction conditions [128,143]. This has facilitated the identification of the formation and evolution of active sites in the reactions.

The similarity between SACs and molecular catalysts has led to predictions that the two systems will exhibit similar catalytic activity in different situations. Sung *et al*. [144] reported that by investigating the reactivity in single-atom Pd/CeO_2_ catalysts, the activity of the Pd SACs increased linearly with the amount of Pd single atoms. *In-situ* Raman characterization showed that an enhancement in the number of Pd single atoms resulted in enhanced reducibility of the CeO_2_ substrate (Fig. 8e). Furthermore, DFT calculations verified that as the density of surface monoatomic Pd increased, the energy of oxygen hole formation decreased, while CO and O_2_ adsorption energies remained unchanged. This further substantiates the hypothesis that the observed increase in catalytic activity of monoatomic Pd is due to the activation of lattice oxygen by Pd. The aforementioned results, when considered alongside pertinent computational chemistry simulations, suggest that the catalytic activity of monoatomic species on reduced oxide substrates can be influenced by modifying the concentration of monoatomic sites. Peng *et al*. [142] have made notable advancements in the study of electrolytic seawater hydrogen production coupled with glycerol oxidation applications. This involved the production of formic acid and hydrogen simultaneously in alkaline seawater via the utilization of Ru-doped CoP_2_ (Ru-CoP_2_) as anode and cathode. *In-situ* Raman spectroscopy was employed to ascertain the alterations in the surface structure of the anode. At 1.35 V during OER, a pair of Raman vibrations manifested, with peaks at 457 and 542 cm^−1^ representing CoOOH (Fig. 8f). Following the disconnection of the circuit during testing at 1.60 V and the introduction of 0.1 M glycerol into the reaction system, the Raman signal of CoOOH exhibited a marked decline (Fig. 8g), indicating that the hydroxide underwent spontaneous reaction with glycerol. Conversely, the absence of Raman peaks for CoOOH across the entire electrochemical potential window of the glycerol oxidation reaction (GOR) suggests that CoOOH does not accumulate on the catalyst surface during the GOR process (Fig. 8h). In conclusion, the introduction of Ru doping has been demonstrated to facilitate a profound restructuring of CoP_2_, resulting in the generation of enhanced Ru-CoOOH active species. This, in turn, has been shown to enhance the electrocatalytic activity for selective glycerol oxidation in an alkaline solution.

Recently, an obvious increase in research activities focusing on catalysts based on hydrogen spillover has been witnessed, largely due to the fact that these catalysts possess fully utilized reaction sites [145,146]. Nevertheless, the precise regulatory mechanism governing the intensity of spillover remains elusive. Wang *et al*. [139] constructed a Ru/NiMoO_4−_*_x_* catalyst that was enriched with O vacancies and investigated the intrinsic correlation between electron supply and the mechanism of hydrogen spillover enhancement. *In-situ* Raman spectroscopy was employed to gain deeper insight into the adsorption process of H*. The typical Raman peaks observed at 978 and 1064 cm^−1^ vibrations at 0–0.15 V are attributed to Mo=O and Ni=O, respectively (Fig. 8i). DFT calculations, when combined with *in-situ* Raman spectroscopy, reveal the spillover of H* from NiMoO_4−_*_x_* to Ru. Concurrently, the presence of oxygen vacancies serves to attenuate the electron supply from Ru to NiMoO_4−_*_x_*, thereby contributing to the dilution of the built-in electric field resistance to hydrogen spillover. This work emphasizes the significant contribution of the H* transfer phase to the process of hydrogen spillover, while simultaneously broadening the scope for the development of HER catalysts applied in seawater. In April 2023, Sun *et al*. [134] reported a synthesis strategy for SASCs with room-temperature ferromagnetism, composed of magnetic atoms replaced within a MoS_2_ host. There are three main types of sulfur atoms surrounding the individual NiMo site, including adjacent S atoms (Ni–S bond length 2.521 Å), adjacent S atoms (Ni–S chain length 2.285 Å) and S electrons located at the center of Mo prisms (Fig. 8j). It is the S atoms that form the spin density optimization at the S active site, and the field-regulated Ni–S electron hybridization process results in the generation of optimal adsorption/desorption energies for the radical intermediates, thereby lowering the reaction potential barriers. In the same month, Zhu *et al*. [147] reported a supported ruthenium catalyst, which was superior to the benchmark platinum catalyst in hydrogen generation reactions under alkaline conditions. Dynamic structural evolution under HER conditions was captured by *in-situ* XAS, which indicated that Ru SAs and metallic Ru clusters were the real active species (Fig. 8k and l). The combination of *in-situ* characterization with theoretical calculations allows for a more integrated grasp of the catalytic mechanism of SACs in the electrolysis of seawater.

As research has progressed, significant advances have been made in the synthesis and characterization of SACs. *In-situ* characterization techniques have contributed to our understanding of the mechanism by which SACs catalyze reactions, the manner in which they interact with reactants, and the manner of evolution of the active sites (e.g. alterations in ligand configuration and movement of metal atoms during the course of a reaction) [148,149]. Nevertheless, due to the limitation of the existing characterization technology, our understanding of the electronic structure of catalysts and their kinetics under actual working conditions is still incomplete. In order to better understand the mechanism and kinetics of SACs in real reaction systems, as well as to facilitate the design of improved SACs, it is necessary to develop more precise *in-situ* devices and computational simulations.

## CHALLENGES AND RESPONSES

The growing global demand for sustainable development and clean energy, along with the drive for environmental policies, has created a unique set of circumstances for the catalysts market, offering unprecedented opportunities [150]. Yadong Li, an academician of the Chinese Academy of Sciences, has indicated that noble metal SACs possess notable advantages in terms of both activity and selectivity in specific reactions. These advantages have been demonstrated in a number of fields, including automobile exhaust purification, ethylene epoxidation and fuel cells. As scientific and technological advancement continues, the preparation method and cost control of SACs will undergo continuous optimization, thereby conferring upon them considerable potential for industrialization.

**Figure 9.**
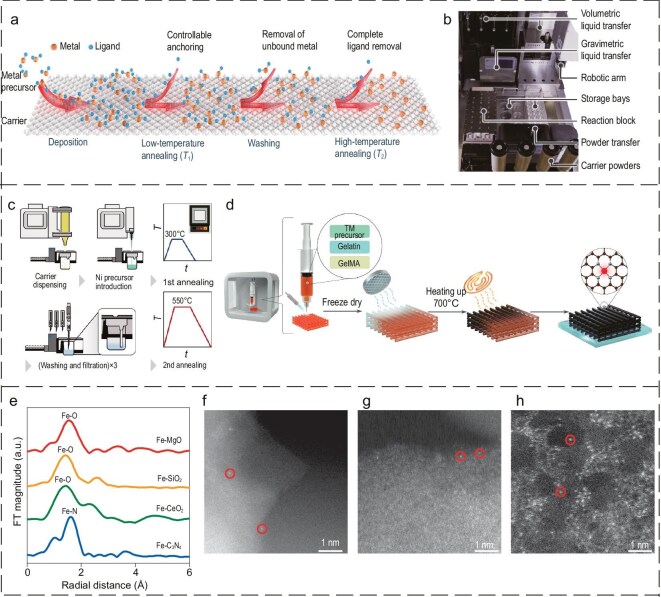
(a) Strategy for the preparation of UHD-SACs. (b) Photograph of the robotic synthesis platform. (c) Flowsheet of the synthesis protocol. (a–c) Reproduced with permission from ref. [151]. Copyright 2021, Springer Nature. (d) Schematic diagram for 3D-printed SACs. Reproduced with permission from ref. [152]. Copyright 2023, Springer Nature. (e) EXAFS-derived radial distribution functions of Fe-MgO, Fe-SiO_2_, Fe-CeO_2_ and Fe-C_3_N_4_. HAADF-STEM image of single-atom (f) Fe-MgO, (g) Fe-SiO_2_ and (h) Fe-C_3_N_4_. Reproduced with permission from ref. [153]. Copyright 2023, Springer Nature.

In order to meet the demand for large-scale reaction systems, the development of a universal method for preparing SACs with high surface metal densities is a necessity for the industrialization of single-atom catalysis. Zhao's team [151] has developed a versatile two-step annealing strategy based on facile wet chemistry for the large-scale synthesis of ultra-high-density SAC (UHD-SACs) libraries. The method initially achieves high metal coverage by selectively anchoring the metal precursor onto the carrier, followed by controlled removal of ligands from the metal precursor to enhance metal-carrier interactions. This is followed by a second annealing at a higher temperature (*T*_2_), which converts the chemisorbed metal precursor into UHD-SACs with a well-defined atomic structure by removing the remaining ligand from the chemically adsorbed metal precursor (Fig. 9a). Furthermore, the method was successfully applied to automated mode preparation (Fig. 9b and c). In addition to the automated mode preparation, the two-step annealing method can be readily scaled up to the kilogram scale due to the straightforward operational process. The reproducibility and properties of the resulting products demonstrate minimal variation, substantiating the method's capacity for precise and effective control. Joe's team [152] reported a general 3D printing synthesis method for directly constructing a library of SACs (Fig. 9d). The method involved mixing printing inks with transition metal precursors and subsequently employing 3D printing for the synthesis of a variety of SACs. The generality of the method was demonstrated by the small change in the atomic dispersion observed with synthetic variations of the central atoms, the loading of the central atoms, the coordination environments and the spatial geometries. Additionally, Li *et al*. [153] put forth a methodology for the direct synthesis of SACs utilizing bulk metals and carriers as precursors. In this method, the bulk metal and carrier were directly mixed and milled in a planetary ball mill with a nitrogen atmosphere. The monoatomic loading and morphology could be readily adjusted by varying the ball milling speed, feed amount and ball milling time. Moreover, the method is not only applicable to the preparation of a wide range of metal-N-C systems but can also be extended to other carriers (e.g. oxides and nitrides) (Fig. 9e–h). Furthermore, the method can be scaled up for industrial production by simply increasing the volume of the ball mill vessel, thereby providing a reference for subsequent industrialized production.

The advancement of synthesis methods, diversification of carrier materials, expansion of application areas, and in-depth theoretical studies and computational simulations has contributed to the emergence of SACs as a promising platform for a multitude of applications across diverse fields [154–158]. While stability and durability remain challenges, in-depth research will facilitate the advancement of SACs through strategies such as improved synthesis methods and optimized carrier materials, thereby promoting their commercial application and large-scale production. Despite the ongoing challenges related to cost and production efficiency, technological advancements and market demand suggest a highly optimistic outlook for the industrial application of SACs.

## SUMMARY AND PROSPECTS

DSE offers a significant advantage because of the abundance and low cost of seawater, eliminating the need to consider the additional costs associated with the desalination process. The majority of current research on direct electrolysis of seawater is conducted at the laboratory level. Further refinements are required in the design and scale-up of new types of electrolysis cells for seawater electrolysis and in the development of suitable catalysts to meet the requirements of hydrogen production on an industrial scale. The latter process could potentially be accelerated by drawing on the experience of the successful industrial application of freshwater electrolysis.

Furthermore, the occurrence of toxicity and side reactions at the catalytic active sites is a consequence of the complex environment in seawater, and the contradiction between the vast reserves of the target substances and their low concentrations remains a significant challenge. The tunability of SACs allows for the optimization of the types of metal atoms, thereby improving the rate of resource conversion. Furthermore, the selectivity of catalysts for the conversion of specific substances can be enhanced by adjusting the coordination environments of single atoms and altering the changes in the Gibbs free energies of the intermediate states of the reaction. This enables the overcoming of the interference of hetero ions in seawater. This paper presents numerous ways for regulating SACs to facilitate the electrolysis of seawater. The study of SACs in seawater systems is of great significance, as it allows for the analysis of the mechanisms of these catalysts under extreme conditions and the discovery of new breakthroughs in the exploitation of seawater resources. The following section presents an overview of the potential applications of SACs in the field of DSE:

- Optimization of SACs for seawater corrosion resistance: Recent advances in materials science have shifted the focus to the design of novel SAC structures tailored for enhanced corrosion resistance in complex seawater environments. This emerging strategy leverages 2D materials (e.g. graphene) and 3D frameworks (e.g. metal-organic frameworks), known for their exceptional corrosion resistance. A critical challenge is the optimization of the interface between the SACs and these carrier structures, ensuring both the stability and the uniform dispersion of single atoms within the catalyst. A key concept is the matching of single atoms with carrier lattices, optimizing electronic interactions at the interface. These interactions influence the binding strength of active sites, thereby improving catalytic activity and long-term stability. As these SACs undergo further refinement, their competitiveness in practical applications, particularly in seawater electrolysis, will be greatly enhanced, offering a new frontier in catalyst design that addresses both performance and durability.
- *In-situ* characterization techniques for dynamic monitoring of SACs in seawater electrolysis: In-depth understanding of SAC performance during seawater electrolysis demands continuous advancements in *in-situ* characterization techniques. XAS plays a crucial role in revealing the evolution of the catalyst's coordination environment and oxidation states of metal atoms in real time. Furthermore, Fourier transform infrared (FTIR) spectroscopy allows for dynamic monitoring of reaction intermediates, offering valuable insights into reaction mechanisms. The convergence of these techniques, such as combining XAS with FTIR or even integrating operando electrochemical techniques, will provide a comprehensive view of SAC behavior during electrolysis. This integrated approach will be pivotal in identifying key structural and chemical changes in SACs, which will ultimately drive the development of more efficient catalysts. Additionally, the discovery of new, more robust *in-situ* techniques, capable of monitoring real-time dynamic changes at both the atomic and molecular levels, will further accelerate research and application in seawater electrolysis.
- Methodologies for large-scale preparation of SACs: The large-scale production of SACs remains a major barrier to the widespread adoption of seawater electrolysis. While significant strides have been made in developing thermal stabilization techniques for SACs, the cost and scalability of production processes still pose challenges. Current preparation methods, including impregnation, vapor-phase deposition, templating, self-assembly and co-precipitation, each offer distinct advantages but also come with inherent limitations in terms of scalability, uniformity and efficiency. To bridge this gap, novel fabrication methods that enhance cost-effectiveness and scalability while maintaining catalyst performance are urgently needed. Integrating green synthesis routes and leveraging biomass-derived precursors could pave the way for more sustainable and cost-efficient production. Moreover, overcoming the limitations of existing methods will allow SACs to play a transformative role in large-scale hydrogen production via seawater electrolysis, driving the transition to cleaner, more sustainable energy sources.

In conclusion, SACs demonstrate considerable potential for the production of hydrogen from seawater electrolysis. As research progresses, it is possible to achieve not only an increase in the hydrogen yield but also a reduction in energy consumption and cost. Furthermore, the tunability of SACs permits their optimization for disparate reaction conditions, thereby enhancing their applicability and stability in practical applications. While there are still some challenges to overcome, including catalyst stability and large-scale preparation, the continuous progress in materials science and nanotechnology bodes well for the future of SACs in the commercial application of electrolysis of seawater, and their potential to make a positive contribution to the development and utilization of renewable energy. Through interdisciplinary collaboration and innovation, it is anticipated that SACs will play an instrumental role in the sustainable advancement of the marine energy sector, and provide effective solutions to environmental problems and the global energy crisis.
